# A scoping review on the characteristics of chronic pain registries

**DOI:** 10.1097/PR9.0000000000001482

**Published:** 2026-08-14

**Authors:** Babina Rani, Babita Ghai, Mayank Gupta, Rajni Sharma

**Affiliations:** a Department of Physical and Rehabilitation Medicine, Post Graduate Institute of Medical Education and Research, Chandigarh, India; b Department of Anaesthesia and Intensive Care, Post Graduate Institute of Medical Education and Research, Chandigarh, India; c Department of Anaesthesiology, All India Institute of Medical Sciences, Rishikesh, Uttarakhand, India; d Department of Paediatrics, Post Graduate Institute of Medical Education and Research, Chandigarh, India

**Keywords:** Chronic pain, Patient registries, Pain measurement, Minimal dataset

## Abstract

Chronic pain represents a major global public health challenge, yet systematic surveillance through patient registries remains unevenly developed. Pain registries offer valuable real-world data to inform clinical practice, research, and policy; however, their global scope, design characteristics, and data elements have not been comprehensively mapped. This scoping review aimed to identify and characterize existing chronic pain registries worldwide by examining their geographic distribution, registry design, clinical focus, data domains, and methodological features, including population characteristics, data collection methods, clinical and patient-reported outcomes, follow-up practices, and governance structures. Multiple electronic databases and registry-specific sources were systematically searched to identify chronic pain registries. A total of 36 chronic pain registries were identified across multiple continents, with detailed dataset information available for 16 registries. Registries were predominantly concentrated in high-income countries, particularly Europe and North America, with minimal representation from low- and middle-income regions. Most registries captured broad chronic pain populations, whereas condition-specific registries primarily focused on headache and migraine and fibromyalgia. Considerable heterogeneity was observed in registry scale, maturity, and data domains. Although pain intensity and patient-reported outcomes were commonly recorded, key elements such as pain mechanism classification, socioeconomic variables, and economic evaluations were inconsistently reported. To conclude, the global chronic pain registry landscape remains fragmented, with substantial geographic and methodological disparities. Harmonized data standards, broader geographic representation, and incorporation of mechanism-based and socioeconomic data are essential to enhance the comparability, translational value, and equity in chronic pain research worldwide.

## Abstract 2

Supplemental Digital Content is Available in the Text.

Global chronic pain registries remain fragmented and concentrated in high-income settings, with inconsistent datasets, limited socioeconomic data, and insufficient standardization hindering comparability and equitable pain surveillance.

## 1. Introduction

Chronic pain represents a significant public health challenge globally, affecting an estimated 8.7% to 64.4% of the population and imposing considerable personal, societal, and economic burdens.^24,58^ Given the complex and multifactorial nature of chronic pain conditions, there is a growing emphasis on improving patient outcomes through more personalized and evidence-based interventions. In this context, pain registries have emerged as a promising strategy, as they systematically collect, analyze, and report patient data to enhance understanding of pain conditions and support improvements in health care delivery.^15,23,38^ According to PubMed's medical subject headings (MeSH), registries are the “systems and processes involved in the establishment, support, management, and operation of registers [sic], eg, disease registers.” Patient-focussed registries serve as crucial tools for health professionals, policymakers, and researchers, facilitating longitudinal evidence generation to inform guidelines, resource allocation, and public health strategies.^43,54^

Recent evidence highlights that inconsistencies in the definition and classification of chronic pain continue to limit the comparability of epidemiological findings. For example, a recent meta-analysis by Steingrímsdóttir et al.^58^ underscored the importance of adopting unified definitions of chronic pain to enhance comparability across studies and improve the reliability of multicentre research.

Further global evidence highlights an urgent need to strengthen chronic pain surveillance systems. Complementing this, large-scale burden-of-disease analyses show a steady rise in both the prevalence and years lived with disability due to chronic pain since 1990, with projections indicating continued increases—especially in high sociodemographic index (SDI) regions and among women and older adults.^72^ Similarly, forecasts based on Global Burden of Disease 2021 data suggest that major chronic pain subtypes, including musculoskeletal and arthritis-related pain, will keep increasing through 2032, with marked socioeconomic and demographic disparities across regions.^71^ Concurrently, opioid prescribing trends over the past 15 years and widening global inequities in chronic pain prevalence, where high SDI regions demonstrate higher documented burdens, whereas low SDI regions face challenges such as underdiagnosis, limited surveillance capacity, and inadequate reporting mechanisms factors that may underestimate the true burden.^7,72^ Such disparities further reinforce the necessity of harmonized, methodologically consistent registry frameworks capable of enabling cross-regional comparisons and improving global chronic pain epidemiology.

High-quality pain registries also play an important role in comparative effectiveness research, quality-improvement initiatives, and real-world assessments of treatment outcomes, providing critical evidence to inform policy decisions and support efficient health care resource allocation.^67^ However, substantial variation in registry architecture, data elements, and methods of data capture continues to limit interoperability and restricts the generalizability of findings across different health care settings. This underscores the need for a structured assessment of existing registries to pinpoint strengths, identify methodological shortcomings, and highlight areas where harmonization is needed.

Despite their potential, the quality and characteristics of pain registries vary widely across countries. Factors such as data completeness, patient centredness, registry governance, and the inclusion of patient-reported outcome measures (PROMs) and their priorities can influence the utility of these databases.^43^ Therefore, a scoping review evaluating the characteristics and quality of pain registries is essential to identify existing strengths, limitations, and areas for improvement. Understanding these aspects can help optimize the use of registries in guiding clinical practice and shaping future research on pain management. A previous systematic review by Freytag et al.^20^ considered pain registries; however, the inclusion was limited to registries published in the German language. In addition, there is a scarcity of reviews assessing the design of pain registries.

The aim of this scoping review was to (1) identify and summarize the key characteristics and core data elements of existing chronic pain registries and (2) critically examine their design and data collection methodologies to inform the development of standardized, comprehensive, and interoperable pain registry frameworks. By synthesizing the available evidence, this review seeks to provide insights into best practices and areas for improvement in the development and utilization of pain registries to enhance patient outcomes and health care efficiency.

## 2. Methodology

Considering the exploratory nature of the review question, we considered scoping review as the best fit to map the available evidence on the topic.^2,36^ An online tool ie, right review validated our choice of the review methodology.^1^ This scoping review was conducted in line with the Arksey and O'Malley framework enhanced by Levac, Colquhoun, and O'Brien for conduct of scoping reviews: identifying the review question, defining eligibility criteria, identifying relevant studies, study selection, charting data, and collating, summarizing, and reporting the results.^2,36^ The review was conducted in adherence to an a priori protocol delineating the review objectives, information sources, inclusion and exclusion criteria, data extraction, and presentation.^49^ In line with the recommendations for scoping reviews, a critical appraisal of the studies was not performed.^2,36,49,62^ The review has been reported as per Preferred Reporting Items for Systematic Reviews and Meta-analyses (PRISMA-ScR) guidelines, and the protocol was registered on the Open Science Framework (osf.io/a9qt2).

### 2.1. Data sources and search strategy

The electronic databases (PubMed, Scopus, Web of Science, CINAHL via EBSCOhost, EMBASE) and various registry portals (Table 1) were systematically searched for the available literature on the registries on chronic pain, published till June 2025. In addition, the reference lists of included studies and the relevant reviews were manually searched for other potential studies for inclusion. Furthermore, an extended internet search was conducted using Google scholar.

**Table 1:** Search strategy details.

	Source/database	Search strategy/terms used	n
Electronic database searches	PubMed	“pain registry” OR “pain registries” OR “chronic pain registry” OR “chronic pain registries” OR “pain database” “pain registry”[All Fields] OR “pain registries”[All Fields] OR “chronic pain registry”[All Fields] OR ((“chronic pain”[MeSH Terms] OR (“chronic”[All Fields] AND “pain”[All Fields]) OR “chronic pain”[All Fields]) AND (“registries”[MeSH Terms] OR “registries”[All Fields] OR “registry”[All Fields] OR “registries”[All Fields])) OR “pain database”[All Fields]	2397
	Scopus	TITLE-ABS-KEY (“pain registry” OR “pain registries” OR “chronic pain registry” OR “chronic pain registries” OR “pain database”)	256
	Embase	“pain registry” OR “pain registries” OR “chronic pain registry” OR “chronic pain registries” OR “pain database”	259
	CNAHL	“pain registry” OR “pain registries” OR “chronic pain registry” OR “chronic pain registries” OR “pain database”	64
	WoS	“pain registry” OR “pain registries” OR “chronic pain registry” OR “chronic pain registries” OR “pain database”	163
	*Total number of records identified*		3139
Registry and clinical trial portals (manual search)	Registry of Patient Registries (RoPR) (https://patientregistry.ahrq.gov)	“pain registry,” “chronic pain,” “registry,” “database”	573
	Australian Register of Clinical Registries (https://www.safetyandquality.gov.au/our-work/indicators-measurement-and-reporting/national-guidance-clinical-quality-registries/australian-register-clinical-registries)		15
	OrphaNet (https://www.orpha.net/)		19
	US Clinical trials registry (https://clinicaltrials.gov)		96
	WHO International Clinical Trials Registry Platform (ICTRP) (https://trialsearch.who.int)		693
	EU Clinical Trials Register (EU-CTR) (https://www.clinicaltrialsregister.eu)		96
	*Total number of records identified*		1492

The databases were searched using terms that included controlled vocabulary and their textwords for “chronic,” “pain,” and “registry,” using the Boolean operators OR and AND. The query was modified independently and grouped using AND “medical,” “clinical,” “database,” “patient,” “data repository.” The detailed search strategy for each database is outlined in Table 1.

### 2.2. Eligibility criteria

The inclusion criteria for this review were prespecified as follows: (1) registries enrolling patients with chronic noncancer pain, (2) studies involving participants aged 18 years or older, and (3) studies explicitly describing the datasets collected within the registry. Registries focusing primarily on cancer-related pain, neuromodulation, or any surgical procedures were excluded. No restrictions were applied regarding language, publication date, or country of origin.

As recommended by Pollock et al.,^52^ the inclusion criteria for this scoping review were defined using the Population–Concept–Context framework. The population of interest comprised adults with chronic noncancer pain. The core concept addressed was pain registries, defined as organized systems collecting standardized data related to pain conditions. No restrictions were applied with respect to health care setting or geographical context.

Articles that mentioned a registry but did not directly describe its dataset were excluded. However, primary publications presenting registry outcomes were further examined to identify any referenced datasets. The registry contact persons, developers, or corresponding authors of related publications were approached to obtain details on data domains and subdomains using a standardized questionnaire provided electronically (Supplementary Table 1, http://links.lww.com/PR9/A433). Emails were sent in 4 rounds, at intervals of approximately 3 to 4 weeks. Reminder emails were issued when no response was received, and additional follow-up was conducted where necessary. Registries for which complete dataset information could not be obtained after 3 to 4 repeated contact attempts were excluded from the analysis.

To meet the criteria for defining a medical data registry, we applied the following registry characteristics: mergeable data, standardized dataset, systematically and prospectively collected data, observations associated over time, and knowledge about patient-related outcomes.^16^

### 2.3. Screening and data extraction

The initial database search was conducted by B.R., supplemented by manual and registry searches to ensure comprehensive coverage. All retrieved studies were imported into EndNote for duplicate removal before proceeding to screening. Title and abstract screening were conducted by 2 independent reviewers using the Rayyan platform, according to predefined eligibility criteria. Studies deemed potentially relevant underwent full-text review for final inclusion.

**Figure 1.**
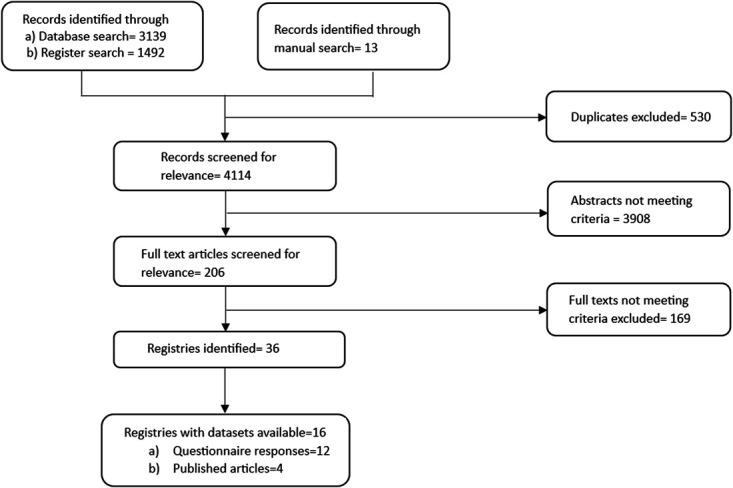
PRISMA flowchart.

Selection of studies and data extraction were performed independently by B.R. and M.G. to minimize bias. Any discrepancies were resolved through consensus discussions, with arbitration by a third reviewer (B.G.) where necessary. Figure 1 describes the flow of the studies for inclusion in the review using PRISMA flowchart.

The data from the included studies/registries were extracted using a customized Microsoft Excel datasheet, based on the following: (1) author and year, (2) geographical location, (3) name of registry, (4) year in which registry was established, (5) setting (primary care/secondary care/tertiary care/community-based/specialised clinics), (6) conditions or disease processes entered, (7) most recently published number of patients recruited and studied, and (8) the patient data collected in the registry (including different domains like demographic details of the patients, clinical details, PROMs, economic evaluation, interventions, and follow-up assessment time points).

## 3. Results

**Figure 2.**
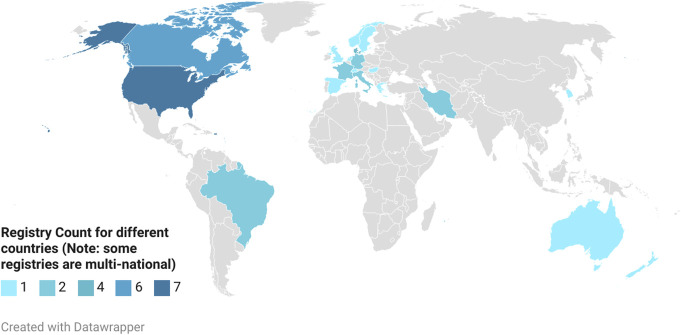
World Map showing registry distribution for different countries.

**Figure 3.**
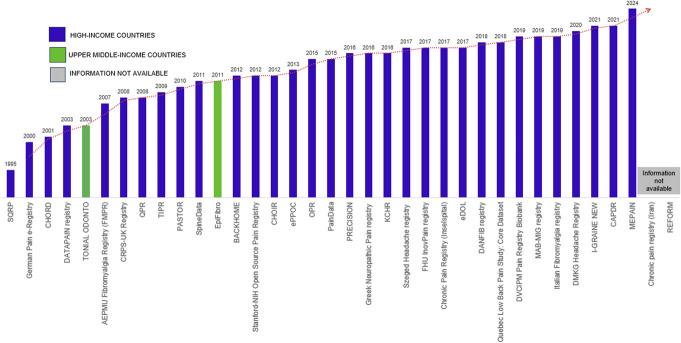
Timeline of chronic pain registry establishment by country income level.

This scoping review identified 36 chronic pain registries^3–5,9,10,13,14,17,18,21,22,25,26,29,31–35,37,39,40,45,47,48,55–57,59,60,63,65,66,68,70^ spanning multiple continents, with the majority concentrated in high-income countries. Registries displayed substantial heterogeneity in their design, scope, and operational frameworks. Geographic distribution analysis showed predominant representation from Europe (n = 17) and North America (n = 13), with limited contributions from the South America (n = 2), Asia (n = 2), Middle East (n = 1), and Oceania (n = 1) (Fig. 2 and Table 2). Few registries had a multinational span (eg, CHOIR, MEPAIN) (Fig. 2). Importantly, no registries were identified from Africa. Notably, 3 registries (EpiFibro, TONIAL ODONTO, and Chronic pain registry, Iran) originated from upper middle-income countries, whereas none were identified from low-income or lower middle-income countries (Fig. 3).

**Table 2:** General details of chronic pain registries.

S.No.	Registry name	Year of establishment	Geographical location (country/region)	Registry setting	Pain conditions covered	No. of patients currently enrolled	Digital/paper-based	Population	Funding	Publicly available website
I. Chronic pain (general) registries										
**1**	**QPR**	**November 2008**	**Province of Quebec, Canada**	**Tertiary care**	**All chronic pains**	**9449 (November 2008–November 2024)**	**Hybrid**	**Adult**	**Government, industry sponsorship**	**Yes**
**2**	**CHOIR**	**2012**	**United States, Canada, other countries**	**Primary care, secondary care, tertiary care, pain clinic**	**All chronic pains**	**100,000–200,000**	**Digital**	**Adult, pediatric**	**Government, academic institution, philanthropy**	**Yes**
**3**	**OPR**	**2015**	**Norway**	**Tertiary care, pain clinic**	**All chronic pains**	**10,000**	**Digital**	**Adult**	**Hospital funded**	**Yes**
**4**	**PainData**	**2015**	**Denmark**	**Tertiary care, rehabilitation centers, pain clinic**	**All chronic pains**	**60,000**	**Digital**	**Adult**	**Funded by department who use registry**	**Yes**
**5**	**Greek Neuropathic Pain registry of PA.RH.SY.A**	**2016**	**Greece**	**Pain and palliative care centers**	**Chronic cancer and noncancer** **Neuropathic Pain**	**2334 (till July 2020)**	**Hybrid**	**Adult**	**Industry sponsorship**	**Yes**
**6**	**eDOL**	**2017**	**France**	**Tertiary care**	**Chronic pain**	**2500**	**Digital**	**Adult**	**Government, academic institution**	**Yes**
**7**	**DVCIPM Pain Registry Biobank**	**2019**	**United States**	**Secondary care, pain clinic**	**All chronic pains**	**500**	**Digital**	**Adult**	**Government**	**Yes**
**8**	**CAPDR**	**2021-03-01**	**Canada**	**Secondary care, tertiary care, rehabilitation centers**	**All chronic pains**	**1991**	**Digital**	**Adult**	**Government**	**Yes**
**9**	**MEPAIN**	**2024**	**Middle East [multinational; single-center data repository]**	**Pain clinic**	**Chronic pain**	**3903 (July 2024–January 2025)**	**Hybrid**	**Adult**	**Academic institution**	**No**
*10*	*SQRP* ^21^	*1995*	*Sweden*	*Pain rehabilitation units*	*Chronic pain*	*19,833 (in 2007)*		*Adult*	*Government and academic institution*	*NA*
*11*	*ePPOC* ^22^	*2013*	*Australia, New Zealand*	*Pain management service*	*Chronic pain*	*NA*	*Digital (software epiCentre)*	*Adult, pediatric*	*Government*	*Yes*
*12*	*Chronic pain registry (Iran)* ^23^	*NA*	*Iran*	*NA*	*Chronic pain*	*NA*	*Digital*	*NA*	*NA*	*NA*
***13***	***German Pain e-Registry (GPeR)*** ^53^	***2000***	***Germany***	***Pain centers***	***Chronic pain***	***260,013 (till 31 December 2019)***	***Digital (web-based application iDocLive)***	***Adult***	***Industry sponsorship***	***NA***
***14***	***DATAPAIN* *registry*** ^39^	***2003***	***Netherlands***	***Pain centre***	***Chronic pain***	***NA***	***NA***	***NA***	***NA***	***NA***
***15***	***Tri-institutional Pain registry*** ^57^	***2009***	***New York, US***	***Pain medicine clinics of WCMC, MSKCC, and HSS.***	***Chronic pain***	***NA***	***Digital***	***NA***	***Government + industry***	***NA***
***16***	***PASTOR*** ^30^	***2010***	***Military Health System, US***	***Pain centre***	***Chronic pain***	***NA***	***Hybrid***	***NA***	***NA***	***Yes***
***17***	***Stanford-NIH Open Source Pain Registry*** ^47^	***2012***	***Stanford, USA***	***Tertiary care outpatient pain clinic***	***Chronic pain***	***2487***	***Digital***	***NA***	***Government***	***NA***
***18***	***Chronic Pain Registry (Inselspital)*** ^27^	***2017/18***	***Switzerland***	***Tertiary care***	***Chronic pain***	***NA***	***Digital (REDCap)***	***Adult***	***NA***	***NA***
**II. Spine registries**										
**19**	**Quebec Low Back Pain Study**	**2018-11-01**	**Canada, Montréal**	**Community-based**	**Low back pain**	**7190**	**Digital**	**Adult**	**Government, academic institution**	**Yes**
*20*	*PRECISION* ^31^	*2016*	*US*	*Community*	*Low back Pain*	*NA*	*Digital (Qualtrics software)*	*Adult*	*Society/foundation*	*Yes*
***21***	***SpineData*** ^24^	***2011***	***Southern Denmark***	***Spine Centre***	***Neck pain, mid-& low back pain***	***36,300 (by early 2015)***	***Digital***	***Adult***	***Private (industry) and public***	***NA***
***22***	***BACKHOME*** ^37^	***2012***	***US***	***NA***	***LBP***	***NA***	***NA***	***NA***	***NA***	***NA***
**III. Headache-migraine registries**										
**23**	**I-GRAINE NEW** ^39^	**2021**	**Italy**	**Secondary care, tertiary care, rehabilitation centers, pain clinic**	**Headache**	**2083**	**Digital**	**Adult**	**Academic institution, industry sponsorship**	**Yes**
***24***	***CHORD*** ^49^	***2001***	***Canada***	***Neurology outpatient clinics***	***Headache & migraine***	***NA***	***Digital***	***Both***	***Industry sponsorship***	***NA***
***25***	***REFORM*** ^50^	***NA***	***Denmark***	***Danish headache center***	***Migraine***	***NA***	***Digital (REDCap)***	***Adult***	***Industry + foundation***	***NA***
***26***	***KCHR*** ^29^	***2016 Sep (v.1)*** ***2018 Oct (v.2)***	***Korea***	***Hospitals (tertiary & secondary referral centres)***	***Cluster headache***	***NA***	***NA***	***Adult***	***NA***	***NA***
***27***	***FHU InovPain* *registry*** ^26^	***2017***	***France***	***Tertiary headache centers***	***Migraine***	***NA***	***NA***	***Adult***	***Industry***	***Yes***
***28***	***Szeged Headache* *registry*** ^36^	***2017***	***Szeged (Hungary)***	***Headache outpatient department at neurologic clinic***	***Migraine***	***412***	***Digital***	***Adult***	***Society + industry sponsorship***	***NA***
***29***	***MAB-MIG* *registry*** ^33^	***2019***	***Spain***	***NA***	***Migraine***	***NA***	***NA***	***NA***	***NA***	***NA***
***30***	***DMKG Headache Registry*** ^35^	***2020***	***Germany***	***Headache centers (clinic-based or private practice)***	***Headache & migraine***	***1351***	***Digital***	***Adult***	***Society + industry sponsorship***	***NA***
**IV. Fibromyalgia registries**										
***31***	***Fibromyalgia Patients Registry (AEPMU)*** ^38^	***2007***	***Canada***	***Pain management centre***	***Fibromyalgia***	***240***	***NA***	***NA***	***NA***	***NA***
***32***	***Brazilian fibromyalgia registry (EpiFibro)*** ^51^	***2011***	***Brazil***	***Public and private health care services***	***Fibromyalgia***	***NA***	***Digital***	***Adult***	***Society***	***NA***
***33***	***DANFIB* *registry*** ^25^	***2018***	***Denmark***	***Hospital***	***FM/CWP***	***NA***	***Digital (REDCap)***	***Adult***	***NA***	***NA***
***34***	***Italian Fibromyalgia registry*** ^28^	***2019***	***Italy***	***Tertiary care rheumatology centres***	***FM***	***6095***	***Digital***	***Adult***	***Society***	***NA***
**V. Other registries**										
**35**	**TONIAL ODONTO**	**2003**	**BRASIL/PARANÁ**	**Pain clinic**	**Musculoskeletal pain, headache, orofacial pain**	**4000**	**Hybrid**	**Adult**	**Consultório Particular**	**No**
***36***	***CRPS-UK Registry*** ^20^	***2008***	***UK***	***NA***	***CRPS***	***618 (2008–2020)***	***Digital***	***Both***	***Government***	***Yes***

Bold coded: Information & dataset obtained from questionnaire responses received; Italic coded: Information & dataset obtained from related publications; Bold italic coded: Information from related publications.

AEPMU, Alan Edwards Pain Management Unit; CAPDR, Canadian Adult Pain Data Registry; CHOIR, Collaborative Health Outcomes Information Registry; CHORD, Canadian Headache Outpatient Registry and Database; CWP, chronic widespread pain; ePPOC, Electronic Persistent Pain Outcomes Collaboration; HSS, Hospital for Special Surgery; KCHR, Korean Cluster Headache Registry; MEPAIN, Middle East Pain Registry; MSKCC, Memorial Sloan Kettering Cancer Center; NA, Information not available; OPR, Oslo University Hospital Pain Registry; QLBPS, Quebec Low Back Pain Study; QPR, Quebec Pain Registry; REFORM, Registry for Migraine; WCMC, Weill Cornell Medical College.

vRegistry attributes were derived from a range of sources, including journal articles, abstracts, annual reports, official registry websites, and, where available, direct correspondence with registry personnel using a standardized study form. In addition, 15 registries (OPR, QPR, CHOIR, Quebec Low Back Pain Study [QLBPS], DVCIPM Pain Registry Biobank, PAINDATA, I-GRAINE NEW, CAPDR, PASTOR, ePPOC, PRECISION, Greek Neuropathic Pain Registry [GrNPR], FHU InovPain Registry, CRPS-UK registry, eDOL) had official websites that provided supplementary information not captured in the published literature.

### 3.1. General characteristics of registries

**Figure 4.**
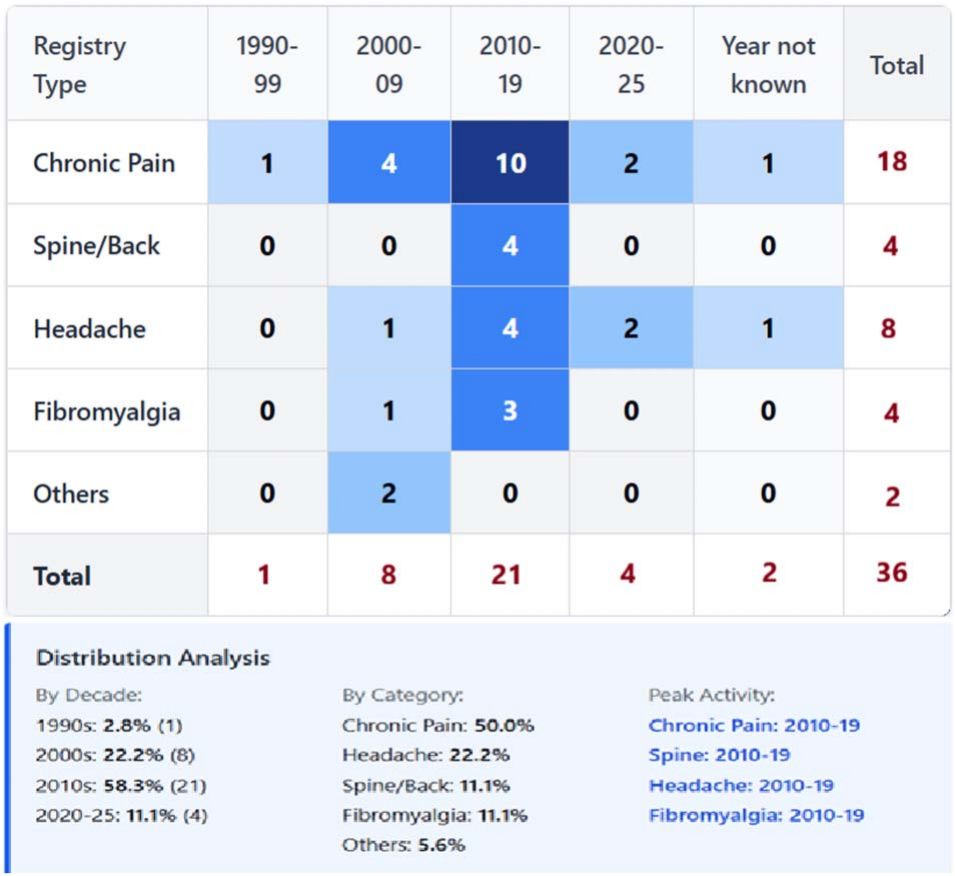
Heatmap for number of registries (decade-wise and specialty-wise). Number of registries established per time period (1990–2025).

The temporal distribution of registry establishment spanned from 1995 to 2024, with a notable increase in registry development after 2010 (Figs. 3 and 4). The Swedish Quality Registry for Pain Rehabilitation (SQRP), established in 1995, represents the longest-running identified registry.^45^ Several registries (n = 2)^3,29^ did not report establishment dates, limiting comprehensive temporal analysis (Table 2).

#### 3.1.1. Registry settings and scope

Registries operated across diverse health care settings, with the majority based in tertiary care centers (n = 12; OPR, QPR, CHOIR, PAINDATA, I-GRAINE NEW, CAPDR, Stanford-NIH Open Source Pain Registry, FHU INOVPAIN, IFMR, DMKG, REFORM, Chronic Pain Registry Inselspital) and pain clinics (n = 14; TONIAL ODONTO, OPR, CHOIR, DVCIPM Pain Registry Biobank, PAINDATA, I-GRAINE NEW, GrNPR, MEPAIN, ePPOC, GPeR, DATAPAIN, TPR, PASTOR, Fibromyalgia Pain Registry AEPMU), whereas smaller numbers functioned within rehabilitation centers (n = 4; PAINDATA, I-GRAINE NEW, CAPDR, SQRP), and secondary care settings (n = 4; CHOIR, DVCIPM Pain Registry Biobank, I-GRAINE NEW, CAPDR) (Table 2). A few registries operated across multiple settings (eg, CHOIR, I-GRAINE NEW, CAPDR). Community-based (n = 2; QLBPS, PRECISION) and primary care registries (n = 1; CHOIR) were notably underrepresented, suggesting limited capture of chronic pain burden at the population level and at primary health care settings. For 4 registries (Chronic Pain Registry Iran, BACKHOME, CRPS-UK, MAB-MIG), setting information was unavailable, highlighting reporting gaps.

The scope of pain conditions covered by the identified registries varied substantially. Eighteen registries addressed chronic pain in a broad, multisite context including one focused specifically on neuropathic pain (GrNPR), whereas others focused on specific pain conditions. Among condition-specific registries, migraine and headache disorders were the most frequently represented (n = 8; I-GRAINE, KCHR, DMKG, InovPain, REFORM, MAB-MIG, CHORD, Szeged), followed by spine and low back pain (n = 4; QLBPS, PRECISION, SpineData, BackHOMe) and fibromyalgia (n = 4; EpiFibro, IFMR, DANFIB, AEPMU). Fewer registries targeted orofacial pain (n = 1; TONIAL ODONTO) and complex regional pain syndrome (n = 1; CRPS-UK) (Table 2).

#### 3.1.2. Enrolment and scalability

The number of patients currently enrolled or most recently reported within the identified registries varied considerably. Large-scale registries, including CHOIR and the German Pain e-Registry (GPeR), reported participant numbers exceeding 100,000, whereas PainData represented the intermediate tier with 10,000 to 100,000 active records. Moderate-scale registries such as OPR, QPR, QLBPS, SQRP, and IFMR reported enrolments between 5,000 and 10,000 participants. Most registries (n = 11) operated with smaller cohorts below 5,000 participants (TONIAL ODONTO, DVCIPM Pain Registry Biobank, I-GRAINE, CAPDR, GrNPR, MEPAIN, Szeged, CHORD, DMKG, Stanford-NIH Open Source Pain Registry, CRPS-UK Registry). Enrolment figures were not available for several registries, limiting a comprehensive evaluation of overall scale (Table 2).

#### 3.1.3. Technological infrastructure and population demographics

Digital data collection platforms predominated, with 23 registries using exclusively digital systems, 6 employing hybrid approaches combining digital and paper-based methods, and 7 with unreported data collection modalities. Data were typically collected through structured self-report questionnaires, capturing items such as pain intensity (eg, numerical rating scales, 0–10), pain duration, pain location (body maps), and functional disability scores. Specific technological platforms included web-based systems (iDocLive for German registries), REDCap-based tools (DANFib, Chronic Pain Registry [Inselspital], REFORM), and custom software such as epiCentre for ePPOC (integrated with REDCap), Qualtrics for PRECISION, and Zigorat for MEPAIN.

The overwhelming majority of registries (n = 24) focused exclusively on adult populations, with only 4 registries (CHOIR, CHORD, ePPOC, CRPS-UK) explicitly including paediatric participants in addition (Table 2).

#### 3.1.4. Funding models

Funding models varied considerably across the 36 registries. Government funding was most prevalent (n = 5; CAPDR, ePPOC, CRPS-UK, DVCIPM Pain Registry Biobank, Stanford-NIH Open Source pain registry), followed by industry sponsorship (n = 4; GrNPR, FHU InovPain, CHORD, German Pain e-Registry), society/trust/foundation (n = 3; Italian fibromyalgia registry, EpiFibro, PRECISION), and academic institutions (n = 2; PAINDATA, MEPAIN). Mixed funding arrangements (n = 11; QPR, QLBPS, SQRP, eDOL, CHOIR, I-GRAINE NEW, Tri-institutional Pain Registry, SpineData, Szeged Headache Registry, DMKG, REFORM) were also observed, encompassing collaborations between government, academic, industry, and societal entities. Hospital-funded (OPR) and private practice (TONIAL ODONTO) models were rare. The funding source was unreported for remaining 9 registries, highlighting gaps in financial transparency and reporting consistency (Table 2).

### 3.2. Registries' core dataset

Detailed dataset information was available for 16 registries. The core datasets for 12 registries were obtained through questionnaire responses (bold coded), whereas datasets for 4 registries were extracted from published articles^3,37,45,60^ (italic coded) (Tables 2–8).

**Table 3:** Demographic elements included in various pain registries.

Registry	Gender	Age	Marital status	Ethnicity/race	Educational level	Employment status/occupation	Height, weight/BMI	Geographical location	Comorbidities/impairments	Social benefits	Disability pension/benefits	Litigation due to pain condition
**TONIAL ODONTO**	**Y**	**Y**	**Y**	**Y**	**N**	**N**	**N**	**N**	**N**	**N**	**N**	**N**
**OPR**	**Y**	**Y**	**Y**	**N**	**Y**	**Y**	**Y**	**Y**	**N**	**Y**	**Y**	**Y**
**QPR**	**Y**	**Y**	**Y**	**Y**	**Y**	**Y**	**N**	**Y**	**Y**	**N**	**Y**	**Y**
**CHOIR**	**Y**	**Y**	**Y**	**Y**	**Y**	**Y**	**Y**	**Y**	**Y**	**Y**	**Y**	**Y**
**QLBPS**	**Y**	**Y**	**N**	**Y**	**Y**	**Y**	**Y**	**Y**	**Y**	**Y**	**Y**	**Y**
**DVCIPM Pain Registry Biobank**	**Y**	**Y**	**Y**	**Y**	**Y**	**Y**	**Y**	**N**	**Y**	**N**	**N**	**N**
**PainData**	**Y**	**Y**	**Y**	**N**	**Y**	**Y**	**Y**	**N**	**Y**	**N**	**N**	**Y**
**I-GRAINE NEW**	**Y**	**Y**	**Y**	**N**	**Y**	**Y**	**Y**	**Y**	**Y**	**N**	**N**	**N**
**CAPDR**	**Y**	**Y**	**N**	**N**	**N**	**N**	**N**	**Y**	**N**	**N**	**N**	**N**
**GrNPR**	**Y**	**Y**	**Y**	**Y**	**Y**	**Y**	**Y**	**Y**	**Y**	**N**	**N**	**N**
**MEPAIN**	**Y**	**Y**	**N**	**Y**	**N**	**N**	**Y**	**Y**	**Y**	**N**	**Y**	**N**
**eDOL**	**Y**	**Y**	**Y**	**N**	**Y**	**Y**	**Y**		**Y**	**Y**	**Y**	**NA**
*ePPOC*	*Y*	*Y*	*NA*	*Y*	*NA*	*Y*	*Y*	*Y*	*Y*	*NA*	*Y*	*NA*
*Chronic Pain Registry (Iran)*	*Y*	*Y*	*Y*	*NA*	*Y*	*Y*	*Y*	*NA*	*Y*	*NA*	*NA*	*NA*
*SQRP*	*Y*	*Y*	*NA*	*NA*	*Y*	*Y*	*NA*	*Y*	*NA*	*NA*	*Y*	*NA*
*PRECISION*	*Y*	*Y*		*Y*	*Y*	*NA*	*NA*	*NA*	*Y*	*NA*	*NA*	*NA*

Bold coded: Dataset obtained from questionnaire responses received; Italic coded: Dataset obtained from related publications.

CAPDR, Canadian Adult Pain Data Registry; CHOIR, Collaborative Health Outcomes Information Registry; ePPOC, Electronic Persistent Pain Outcomes Collaboration; GrNPR, Greek Neuropathic Pain Registry; MEPAIN, Middle East Pain Registry; N, Not included; NA, Information not available; OPR, Oslo University Hospital Pain Registry; PRECISION, Pain Registry for Epidemiological, Clinical, and Interventional Studies and Innovation; QLBPS, Quebec Low Back Pain Study; QPR, Quebec Pain Registry; SQRP, Swedish Quality Registry For Pain Rehabilitation; Y, Included.

**Table 4:** Pain/symptom examination elements included in various pain registries.

Registry	Pain location	Onset/precipitating event	Pain characteristics							Pain impact/interference	Pain mechanism	Diagnosis
			Duration	Frequency	Intensity	Quality	Diurnal variation	AF/RF	Others			
**TONIAL ODONTO**	**Y**	**Y**	**Y**	**Y**	**Y**	**Y**	**Y**	**Y**	**—**	**Y**	**Y**	**Y**
**OPR**	**Y**	**Y**	**Y**	**N**	**Y**	**N**	**Y**	**N**	**—**	**N**	**N**	**Y**
**QPR**	**Y**	**Y**	**Y**	**Y**	**Y**	**Y**	**N**	**N**	**—**	**Y**	**N**	**Y**
**CHOIR**	**Y**	**Y**	**Y**	**Y**	**Y**	**Y**	**N**	**Y**	**—**	**Y**	**Y**	**Y**
**QLBPS**	**Y**	**N**	**Y**	**Y**	**Y**	**N**	**N**	**N**	**—**	**Y**	**N**	**N**
**DVCIPM Pain Registry Biobank**	**Y**	**Y**	**Y**	**N**	**Y**	**Y**	**N**	**N**	**—**	**Y**	**Y**	**Y**
**PainData**	**Y**	**N**	**Y**	**Y**	**Y**	**N**	**N**	**N**	**—**	**Y**	**N**	**Y**
**I-GRAINE NEW**	**Y**	**Y**	**Y**	**Y**	**Y**	**Y**	**N**	**Y**	**—**	**N**	**N**	**Y**
**CAPDR**	**Y**	**Y**	**Y**	**Y**	**Y**	**Y**	**N**	**N**	**—**	**Y**	**N**	**N**
**GrNPR**	**Y**	**Y**	**Y**	**Y**	**Y**	**N**	**N**	**Y**	**Consumption habits**	**Y**	**Y**	**Y**
**MEPAIN**	**Y**	**Y**	**Y**	**Y**	**Y**	**Y**	**Y**	**Y**	**—**	**Y**	**Y**	**Y**
**eDOL**	**Y**	**N**	**Y**	**N**	**Y**	**Y**	**N**	**N**	**N**	**Y**	**N**	**Y**
*ePPOC*	*Y*	*Y*	*Y*	*Y*	*Y*	*NA*	*NA*	*NA*	*NA*	*Y*	*NA*	*NA*
*Chronic Pain Registry (Iran)*	*Y*	*Y*	*Y*	*Y*	*Y*	*Y*	*Y*	*Y*	*Consumption habits*	*Y*	*Y*	*Y*
*SQRP*	*Y*	*NA*	*Y*	*NA*	*Y*	*NA*	*NA*	*NA*	*NA*	*Y*	*Y*	*Y*
*PRECISION*	*NA*	*NA*	*NA*	*NA*	*Y*	*NA*	*NA*	*NA*	*NA*	*Y*	*NA*	*NA*

Bold coded: Dataset obtained from questionnaire responses received; Italic coded: Dataset obtained from related publications.

CAPDR, Canadian Adult Pain Data Registry; CHOIR, Collaborative Health Outcomes Information Registry; ePPOC, Electronic Persistent Pain Outcomes Collaboration; GrNPR, Greek Neuropathic Pain Registry; MEPAIN, Middle East Pain Registry; N, Not included; NA, Information not available; OPR, Oslo University Hospital Pain Registry; PRECISION, Pain Registry for Epidemiological, Clinical, and Interventional Studies and Innovation; QLBPS, Quebec Low Back Pain Study; QPR, Quebec Pain Registry; SQRP, Swedish Quality Registry For Pain Rehabilitation; Y, Included.

**Table 5:** Patient reported outcome measures included in various pain registries.

Registry	Pain intensity	Disability	HR-QoL	Psych. distress	Perceived self-efficacy	Fatigue	Impact on sleep	Pain catastrophizing	Self-report of generic health and wellbeing	Injustice experienced by patients	Any other
**TONIAL ODONTO**	**Y**	**Y**	**Y**	**Y**	**Y**	**Y**	**Y**	**Y**	**Y**	**Y**	**—**
**OPR**	**Y**	**Y**	**Y**	**Y**	**N**	**N**	**Y**	**Y**	**Y**	**Y**	**Personality questionnaire, bodily distress syndrome**
**QPR**	**Y**	**Y**	**Y**	**Y**	**N**	**N**	**Y**	**Y**	**Y**	**N**	**—**
**CHOIR**	**Y**	**Y**	**Y**	**Y**	**Y**	**Y**	**Y**	**Y**	**Y**	**Y**	**Multiple additional PROMIS measures of physical, psychological, social functioning**
**QLBPS**	**Y**	**Y**	**Y**	**Y**	**N**	**Y**	**Y**	**Y**	**Y**	**N**	**PROMIS measures**
**DVCIPM Pain Registry Biobank**	**Y**	**N**	**Y**	**Y**	**Y**	**Y**	**Y**	**Y**	**Y**	**N**	**Interference, physical function, depression, anxiety, anger, alcohol use**
**PainData**	**Y**	**Y**	**Y**	**Y**	**Y**	**Y**	**Y**	**Y**	**Y**	**N**	**—**
**I-GRAINE NEW**	**Y**	**Y**	**N**	**N**	**N**	**N**	**Y**	**N**	**N**	**N**	**—**
**CAPDR**	**Y**	**Y**	**Y**	**Y**	**N**	**N**	**Y**	**N**	**N**	**N**	**—**
**GrNPR**	**Y**	**Y**	**N**	**N**	**N**	**Y**	**Y**	**N**	**N**	**Y**	**—**
**MEPAIN**	**Y**	**Y**	**N**	**N**	**N**	**N**	**N**	**N**	**N**	**N**	**—**
**eDOL**	**Y**	**Y**	**Y**	**Y**	**Y**	**Y**	**Y**	**Y**	**Y**	**N**	**PROMIS 29 + 2**
*ePPOC*	*Y*	*Y*	*Y*	*Y*	*Y*	*NA*	*NA*	*Y*	*NA*	*Y*	*—*
*Chronic pain registry (Iran)*	*Y*	*NA*	*Y*	*Y*	*NA*	*NA*	*Y*	*NA*	*NA*	*NA*	*Laboratory and Imaging tests*
*SQRP*	*Y*	*Y*	*Y*	*Y*	*Y*	*NA*	*NA*	*NA*	*Y*	*NA*	*CPAQ, TSK, LiSat-11, MPI*
*PRECISION*	*Y*	*Y*	*Y*	*Y*	*Y*	*Y*	*Y*	*Y*	*NA*	*Y*	*PROMIS, PSQ, Communication Behavior Questionnaire*

Bold coded: Dataset obtained from questionnaire responses received; Italic coded: Dataset obtained from related publications.

ASC‐12, 12‐item Allodynia Symptom Checklist; CAPDR, Canadian Adult Pain Data Registry; CHOIR, Collaborative Health Outcomes Information Registry; CPAQ, Chronic Pain Acceptance Questionnaire; ePPOC, Electronic Persistent Pain Outcomes Collaboration; FIQR, revised Fibromyalgia Impact Questionnaire; GrNPR, Greek Neuropathic Pain Registry; HIT-6, Headache Impact Test-6; LiSat-11, Life Satisfaction Questionnaire with 11 items; MEPAIN, Middle East Pain Registry; MIDAS, Migraine Disability Assessment; ModFAS, modified Fibromyalgia Assessment Status; MPI, Multidimensional Pain Inventory; N, Not included; NA, Information not available; OPR, Oslo University Hospital Pain Registry; PDQ, Pain Detect Questionnaire; PDS, Polysymptomatic Distress Scale; PRECISION, Pain Registry for Epidemiological, Clinical, and Interventional Studies and Innovation; PROMIS, Patient-Reported Outcomes Measurement Information System; PSQ, Pain Sensitivity Questionnaire; QLBPS, Quebec Low Back Pain Study; QPR, Quebec Pain Registry; SQRP, Swedish Quality Registry For Pain Rehabilitation; SSS, symptom severity index; TSK, Tampa Scale of Kinesiophobia; WHODAS 2.0, World Health Organization Disability Assessment Schedule 2.0; Y, Included.

**Table 6:** Pain treatments included in various registries.

Registry	Current pharmacological pain treatment	Side effects of current pharmacological pain treatment	Past pharmacological pain treatment	Current and past nonpharmacological pain treatments	Type of health care professionals consulted	Surgical intervention
**TONIAL ODONTO**	**N**	**Y**	**Y**	**Y**	**Y**	**N**
**OPR**	**Y**	**N**	**N**	**N**	**Y**	**N**
**QPR**	**Y**	**Y**	**Y**	**Y**	**Y**	**Y**
**CHOIR**	**Y**	**Y**	**Y**	**Y**	**N**	**Y**
**QLBPS**	**Y**	**N**	**Y**	**Y**	**N**	**Y**
**DVCIPM Pain Registry Biobank**	**Y**	**N**	**Y**	**Y**	**N**	**Y**
**PainData**	**Y**	**N**	**Y**	**Y**	**N**	**Y**
**I-GRAINE NEW**	**Y**	**Y**	**Y**	**Y**	**Y**	**Y**
**CAPDR**	**N**	**N**	**N**	**N**	**N**	**N**
**GrNPR**	**Y**	**Y**	**Y**	**Y**	**Y**	**Y**
**MEPAIN**	**Y**	**N**	**N**	**Y**	**N**	**Y**
**eDOL**	**Y**	**N**	**Y**	**Y**	**Y**	**Y**
*ePPOC*	*Y*	*NA*	*NA*	*Y*	*Y*	*NA*
*Chronic Pain Registry (Iran)*	*Y*	*Y*	*Y*	*Y*	*NA*	*NA*
*SQRP*	*NA*	*NA*	*NA*	*Y*	*Y*	*NA*
*PRECISION*	*Y*	*Y*	*NA*	*Y*	*NA*	*NA*

Bold coded: Dataset obtained from questionnaire responses received; Italic coded: Dataset obtained from related publications.

CAPDR, Canadian Adult Pain Data Registry; CHOIR, Collaborative Health Outcomes Information Registry; ePPOC, Electronic Persistent Pain Outcomes Collaboration; GrNPR, Greek Neuropathic Pain Registry; MEPAIN, Middle East Pain Registry; N, Not included; NA, Information not available; OPR, Oslo University Hospital Pain Registry; PRECISION, Pain Registry for Epidemiological, Clinical, and Interventional Studies and Innovation; QLBPS, Quebec Low Back Pain Study; QPR, Quebec Pain Registry; SQRP, Swedish Quality Registry For Pain Rehabilitation; Y, Included.

**Table 7:** Follow-up assessments included in various pain registries.

Registry	Follow-up interval	Follow-up assessments
**TONIAL ODONTO**	**1 mo, 3 mo**	**Pain examination**
**OPR**	**12 mo**	**PROMs, PGIC**
**Pain Registry Biobank**	**1–3 mo 6–9 mo, 12–15 mo, yearly**	**Same as at baseline**
**QPR**	**6 mo, 12 mo, 24 mo**	**Pain examination, PROMs, pharmacological and nonpharmacological treatments, PGIC**
**CHOIR**	**Follow-up assessments tied to clinic visits. For specific projects, have follow up at defined periods depending on project**	**Pain examination, PROMs, economic evaluation**
**QLBPS**	**3 mo, 6 mo, 12 mo, 24 mo, 60 mo, 120 mo**	**Pain examination, PROMs**
**DVCIPM Pain Registry Biobank**	**3 mo, 6 mo, 12 mo, yearly**	**Pain examination, PROMs**
**PainData**	**After treatment**	**PROMs, PGIC**
**I-GRAINE NEW**	**3 mo, 6 mo**	**Pain examination, PROMs**
**CAPDR**	**3 mo, 6 mo, 12 mo, 9 mo**	**Pain examination, PROMs**
**GrNPR**	**1 mo, 2 mo**	**Pain examination**
**MEPAIN**	**1month, 6 mo**	**VAS, any adverse events**
**eDOL**	**1 mo, 3 mo**	**Barometers every week, body maps every month, PROMs every trimester**
*ePPOC*	*End of Rx, 3–6 m after end of Rx, within the Rx period if active Rx exceeds 6m*	*PROMs, ePPOC Patient Impression of Change (ePIC) assessment tool*
*SQRP*	*After rehab, after 1 year*	*PROMs*
*PRECISION*	*Quarterly*	*PROMs*
	*4 wk*	*Drug adverse events*
*Chronic Pain Registry (Iran)*	*NA*	*NA*

Bold coded: Dataset obtained from questionnaire responses received; Italic coded: Dataset obtained from related publications.

CAPDR, Canadian Adult Pain Data Registry; CHOIR, Collaborative Health Outcomes Information Registry; ePPOC, Electronic Persistent Pain Outcomes Collaboration; GrNPR, Greek Neuropathic Pain Registry; MEPAIN, Middle East Pain Registry; N, Not included; NA, Information not available; OPR, Oslo University Hospital Pain Registry; PRECISION, Pain Registry for Epidemiological, Clinical, and Interventional Studies and Innovation; QLBPS, Quebec Low Back Pain Study; QPR, Quebec Pain Registry; SQRP, Swedish Quality Registry For Pain Rehabilitation; Y, Included.

**Table 8:** Economic evaluations included in various registries.

Registry	Economic evaluation performed	Economic aspects assessed
**TONIAL ODONTO**	**N**	**—**
**OPR**	**N**	**—**
**Pain Registry Biobank**	**N**	**—**
**QPR**	**N**	**—**
**CHOIR**	**Y**	**Amount and source of income**
**QLBPS**	**N**	**—**
**DVCIPM Pain Registry Biobank**	**N**	**—**
**PainData**	**N**	**—**
**I-GRAINE NEW**	**Y**	**Cost of treatments, cost of investigations**
**CAPDR**	**N**	**—**
**GrNPR**	**N**	**—**
**MEPAIN**	**N**	**—**
**eDOL**	**N**	**—**
*ePPOC*	*Y*	*Impact of pain on work productivity and employment; impact of pain on school and carer work productivity and employment*
*SQRP*	*Y*	*Work absence*
*PRECISION*	*NA*	*NA*
*Chronic Pain Registry (Iran)*	*NA*	*NA*

Bold coded: Dataset obtained from questionnaire responses received; Italic coded: Dataset obtained from related publications.

CAPDR, Canadian Adult Pain Data Registry; CHOIR, Collaborative Health Outcomes Information Registry; ePPOC, Electronic Persistent Pain Outcomes Collaboration; GrNPR, Greek Neuropathic Pain Registry; MEPAIN, Middle East Pain Registry; N, Not included; NA, Information not available; OPR, Oslo University Hospital Pain Registry; PRECISION, Pain Registry for Epidemiological, Clinical, and Interventional Studies and Innovation; QLBPS, Quebec Low Back Pain Study; QPR, Quebec Pain Registry; SQRP, Swedish Quality Registry For Pain Rehabilitation; Y, Included.

#### 3.2.1. Demographic data elements

All registries reporting demographic data consistently captured core variables such as age and gender, forming the foundation for participant characterization (Table 3). Broader demographic domains, including marital status (n = 10), educational level (n = 12), and employment or occupational status (n = 12), were also commonly recorded, reflecting recognition of socioeconomic influences on pain outcomes. Anthropometric parameters (like height/weight/BMI) were frequently documented (n = 11), supporting analyses of obesity and its association with chronic pain, whereas geographic location (n = 10) provided valuable context for evaluating health care accessibility. In contrast, ethnicity or racial background (n = 9) was captured less consistently, indicating persisting gaps in demographic comprehensiveness across registries. Conversely, comorbidity information (n = 12) was more consistently available, facilitating exploration of the close association between chronic pain and multimorbidity. Data on socioeconomic indicators were limited, with disability pension or benefits (n = 8), litigation status (n = 5), and social benefits (n = 4) infrequently reported, despite their policy and clinical relevance (Table 3).

#### 3.2.2. Pain assessment components

Pain location mapping was nearly universal (n = 15), employing body diagrams, anatomical region checklists, or narrative descriptors (eg, anterior/posterior body maps with shading of affected areas, or standardized body region checklists such as those used in the Brief Pain Inventory) (Table 4). Temporal characteristics demonstrated variable capture, with pain duration (n = 15), onset or precipitating events (n = 11), and frequency (n = 11) being most commonly documented. Collectively, these parameters facilitate differentiation between acute-on-chronic exacerbations, continuous pain, and intermittent pain patterns, supporting more precise clinical characterization.

Pain intensity assessment was ubiquitous among registries reporting pain examination details (n = 16), although specific measurement scales varied (eg, Numerical Rating Scale [NRS] 0 to 10, Visual Analogue Scale [VAS]). Pain quality descriptors (eg, burning, stabbing, throbbing, aching, as captured by tools such as the McGill Pain Questionnaire or the PainDETECT) were documented in 9 registries, whereas diurnal variation received limited attention (n = 4). Aggravating and relieving factors (eg, physical activity, rest, heat/cold application, posture, stress) were systematically captured in only 6 registries.

Pain interference (n = 14) was commonly captured across registries, underscoring its relevance in assessing functional impact. Pain mechanism classification (n = 7), encompassing nociceptive, neuropathic, and nociplastic subtypes (eg, clinical classification using the IASP taxonomy, or screening tools such as painDETECT or DN4 for neuropathic components), was explicitly reported in a smaller subset, reflecting the emerging adoption of mechanism-based pain frameworks. Diagnostic coding (n = 12) was also documented; however, the use of standardized classification systems, such as ICD codes or the IASP taxonomy, remained inconsistent across registries.

#### 3.2.3. Patient-reported outcome measures

Patient-reported outcome measures demonstrated the greatest level of standardization across registries. Pain intensity scales were universally applied among those capturing PROM data (eg, NRS, VAS, or the Patient-Reported Outcomes Measurement Information System [PROMIS] Pain Intensity scale), forming the core component of outcome assessment. Disability measures and health-related quality of life tools (n = 14, 13, respectively) were widely employed (eg, Pain Disability Index [PDI], Roland Morris Disability Questionnaire [RMDQ], EQ-5D, SF-36, or PROMIS Physical Function), capturing both functional limitations and the broader impact of chronic pain on overall well-being. Psychological distress screening, encompassing depression, anxiety, and general mental health (n = 13) (eg, Patient Health Questionnaire [PHQ-9], Generalized Anxiety Disorder scale [GAD-7], Hospital Anxiety and Depression Scale [HADS], or the Depression Anxiety Stress Scales [DASS]), was also consistently integrated, underscoring the biopsychosocial orientation of contemporary pain registries (Table 5).

Assessment of psychosocial and functional domains demonstrated considerable variability across registries. Sleep impact (n = 13) was the most frequently reported parameter (eg, Insomnia Severity Index [ISI], Pittsburgh Sleep Quality Index [PSQI], or single-item sleep interference questions from the BPI), reflecting recognition of sleep disturbance as a key component of chronic pain burden. Pain catastrophizing (n = 10), a central psychological construct linked to pain chronicity and maladaptive coping, was also commonly included (eg, Pain Catastrophizing Scale [PCS]). Generic health or well-being (n = 9) and fatigue (n = 8) were regularly documented, indicating attention to broader functional outcomes. In contrast, measures of self-efficacy (n = 8) and perceived injustice or inequity (n = 6), such as those derived from the Injustice Experience Questionnaire, were less frequently incorporated, highlighting underrepresentation of important psychosocial dimensions in chronic pain evaluation.

Specialized instruments varied by registry focus. Condition-specific registries employed targeted assessments: fibromyalgia registries used the Fibromyalgia Impact Questionnaire (FIQ/FIQR) (capturing domains such as pain, fatigue, stiffness, and physical function specific to fibromyalgia, headache registries incorporated the Headache Impact Test [HIT-6] and Migraine Disability Assessment [MIDAS]), and spine registries included condition-specific disability scales.

#### 3.2.4. Treatment documentation

Current pharmacological treatments (n = 13) were the most frequently documented across registries, followed by past pharmacological treatments (n = 10), which facilitated assessment of longitudinal treatment trajectories. Nonpharmacological interventions (n = 14) (eg, physiotherapy, cognitive behavioural therapy, acupuncture, mindfulness-based stress reduction) were similarly well represented, reflecting recognition of multimodal pain management approaches. Documentation of surgical history (n = 9) provided valuable clinical context. In contrast, adverse medication effects (n = 7) and health care professional consultation patterns (n = 8) were reported less consistently, indicating underrepresentation of key domains relevant to safety monitoring and care pathway evaluation (Table 6).

Treatment documentation granularity varied substantially. Some registries captured detailed medication information including specific agents, dosages, and titration schedules, whereas others employed broader categorical classifications. Nonpharmacological treatment documentation similarly ranged from detailed intervention specifications to general categorical descriptions.

#### 3.2.5. Longitudinal follow-up protocols

Follow-up assessment protocols demonstrated considerable heterogeneity. The included registries implemented structured follow-up schedules, with intervals ranging from 1 month to 10 years. Common follow-up time points included 3, 6, and 12 months (n = 9, 8, and 7, respectively) typically readministering core outcome measures such as pain intensity, disability, and quality of life at each time point to assess longitudinal trajectories. The QLBPS implemented the most extended follow-up protocol (120 months), whereas some registries tied follow-up assessments to clinical visit schedules or post-treatment visit rather than predetermined intervals (eg, CHOIR, PainData). Follow-up assessments typically encompassed repeated pain examinations, PROM administration, and Patient Global Impression of Change (PGIC) evaluation (Table 7).

#### 3.2.6. Economic evaluation components

Economic evaluation represented the most underdeveloped domain across chronic pain registries. Only 4 registries (CHOIR, I-GRAINE NEW, ePPOC, SQRP) explicitly incorporated economic assessments (Table 8). The CHOIR registry assessed income sources and amounts. The I-GRAINE NEW registry documented treatment and investigation costs, whereas ePPOC recorded the productivity loss in both adult and paediatric cohort. Work absence was documented by SQRP. The limited economic data collection represents a critical gap, given chronic pain's substantial societal burden through health care utilization, productivity loss, and disability costs. Without systematic economic evaluation, registries provide incomplete evidence for health policy decision making and resource allocation.

## 4. Discussion

This scoping review identified 36 chronic pain registries spanning multiple continents, revealing substantial heterogeneity in design, scope, and operational frameworks that underscores the evolving but immature state of global chronic pain surveillance infrastructure. The findings highlight both progress and persistent challenges in establishing comprehensive, high-quality pain registries capable of informing clinical practice, policy development, and research.

A pronounced concentration of registries was observed in high-income countries, particularly in Europe and North America, with minimal or absent representation from low- and middle-income regions, underscoring the global inequity in pain data infrastructure and the pressing need to expand registry development beyond resource-rich settings. This geographic imbalance likely reflects disparities in research infrastructure, funding mechanisms, and the resource-intensive nature of registry establishment and maintenance. The absence of registries from Africa and most of Asia is particularly concerning and highlights a critical gap in global chronic pain data infrastructure, given the substantial burden of untreated chronic pain in these regions^27,28,41^ and the growing recognition of pain as a major global public health issue.^24^ The paradox wherein regions facing the greatest pain burden lack systematic data collection mechanisms perpetuates inequities in evidence generation and policy formulation. Strengthening registry capacity in such contexts is, therefore, critical to inform equitable health planning, prevention, and management strategies.

The acceleration of pain registry establishment after 2010 indicates the relatively recent emergence of chronic pain registry infrastructure globally, in contrast to other medical domains such as cardiology, where large-scale national and international registries were established as early as the 1950s and expanded exponentially from the 1980s onward.^12^ In 1995, the Swedish Rehabilitation Association first established a national qualitative registry system (SQRP) aimed at rehabilitating patients with chronic musculoskeletal pain and assessing rehabilitation program impact through patient-reported outcomes. Despite this early pioneer, most identified registries have been operational for less than a decade, which is insufficient time to demonstrate the sustained quality improvements and outcomes that mature registry systems can achieve.

The predominance of tertiary care centers and specialized pain clinics introduces a clear capture bias. Although such settings offer clinically rich datasets and facilitate detailed assessment of complex cases, they overrepresent a small subset of the chronic pain population. The Quebec Pain Registry (QPR) explicitly acknowledged this limitation, emphasizing that its findings reflect only patients referred to tertiary clinics and cannot be generalized to those managed in primary or secondary care.^10^ Given that most chronic pain is treated in primary care, where early intervention can mitigate chronicity, the scarcity of registries in primary, community, and rehabilitation settings hampers understanding of population-level burden, early-stage trajectories, and preventive management. Future registry initiatives should, therefore, ensure broader inclusion across the care continuum to enable comprehensive characterization of chronic pain from onset through long-term outcomes.

Overall, the observed distribution of pain registries reveals important structural and thematic patterns in global chronic pain research. Although a small number of large, digitally integrated registries capture substantial participant volumes, the majority operate at a relatively modest scale, reflecting heterogeneity in registry maturity, scope, and available resource capacity. Although most identified registries adopted a broad scope and encompassed multiple chronic pain conditions, notable imbalances emerged among condition-specific registries. Within this subgroup, there was a stronger research emphasis on headache and migraine, musculoskeletal, and fibromyalgia registries, with comparatively limited representation of neuropathic and orofacial pain conditions. This imbalance underscores both thematic and infrastructural gaps in current registry landscapes. Notably, the widespread adoption of digital data-collection platforms across registries aligns with a broader global shift toward electronic health record integration, suggesting growing readiness for scalable, interoperable pain data systems, albeit unevenly realized across regions and pain conditions.

### 4.1. Dataset standardization and quality considerations

Defining a standardized minimum dataset is a foundational step in developing chronic pain registries. This review revealed marked variability in core data elements, underscoring the absence of internationally endorsed standards. A harmonized dataset is essential to ensure consistency, comparability, and interoperability across institutions and countries^23,54^; however, such consensus remains lacking in pain medicine, resulting in inconsistent data capture and variable quality across registries. The PRECISION registry, for example, incorporated the *Minimum Dataset recommended by the National Institutes of Health Task Force on Research Standards for Chronic Low Back Pain* for its low back pain cohort, representing an important step toward methodological alignment. In addition, it collected blood and saliva samples to determine patient genotypes for drug metabolism, thereby extending registry utility beyond clinical phenotyping to genomic characterization.^37^

Prior reviews of chronic pain registries corroborate and contextualize the present findings of selective sampling and methodological heterogeneity. Narrative and systematic syntheses consistently report that most registries originate in specialized pain centers, yielding clinically detailed yet nonrepresentative patient cohorts, and demonstrate wide variability in scope, data structure, and granularity.^20,42,64^ The review by Freytag and colleagues, although valuable, was limited to German-language registries and, therefore, lacked broader international representation. These analyses further highlight inconsistent adoption of mechanism-based pain classification, psychosocial constructs, and standardized patient-reported outcome frameworks, reinforcing the need for harmonized core datasets to enable cross-registry comparison and comparative-effectiveness research. Methodological reviews and registry evaluations similarly recognize the potential of registries to enhance quality improvement and real-world evidence generation, while cautioning that heterogeneity in design, data completeness, accuracy, and the predominance of specialist-based data collection constrain generalizability and population-level inference.^6,46^ Collectively, these evaluations underscore the need for international consensus on minimal data elements, interoperable digital infrastructures, and transparent governance frameworks to enhance the utility of pain registries for clinical care, research, and health-system planning.

Demographic fundamentals such as age and gender were consistently captured across registries, as were basic pain descriptors including location and intensity. However, key socioeconomic indicators—such as disability benefits, litigation status, and social support—were rarely recorded, despite chronic pain's well-documented impact on work productivity, disability, and social participation.^53^ Previous studies demonstrate that individuals with chronic pain frequently experience marked psychosocial and functional impairment,^8,11^ underscoring the importance of datasets that extend beyond biomedical variables to encompass socioeconomic and psychosocial determinants of pain outcomes.

Pain mechanism classification, distinguishing nociceptive, neuropathic, and nociplastic types, was explicitly documented in only a few registries, despite growing consensus on the clinical utility of mechanism-based approaches.^44^ This limited adoption underscores a disconnect between evolving pain science and registry implementation, reinforcing the need for harmonized data standards to enhance consistency, interpretability, and translational value.

Patient-reported outcome measures constituted the most standardized domain across chronic pain registries, with pain intensity scales nearly universally applied. However, assessment of specialized psychosocial constructs, such as self-efficacy and perceived injustice, remained limited despite their well-established prognostic and therapeutic relevance.^30,69^ Previous reviews have similarly highlighted that, although registry initiatives have increasingly incorporated PROMs, the inconsistent application of validated multidimensional tools undermines cross-registry comparability and analytic robustness.^42,64^ The PROMIS framework provides a comprehensive and standardized structure encompassing pain interference, fatigue, emotional distress, and physical function, yet its adoption remains heterogeneous. Registries such as CHOIR, Stanford–NIH Open-Source Pain Registry, Pain Registry Biobank, PAINDATA, PASTOR, BACKHOME, and PRECISION have integrated PROMIS domains to varying degrees, extending coverage to physical, psychological, and social functioning. Among these, CHOIR exemplifies best practice through its extensive, multidimensional PROMIS battery aligned with the biopsychosocial model of pain. Nonetheless, the uneven implementation of PROMIS measures continues to constrain data harmonization and the potential for large-scale comparative research. The inconsistent inclusion of domains such as sleep disturbance, fatigue, and pain catastrophizing, which are the key contributors to pain persistence, further indicates a lingering predominance of biomedical assessment frameworks over holistic, biopsychosocial approaches in registry design.

The observed variability in treatment-related data capture across chronic pain registries underscores fundamental differences in registry objectives and clinical integration. The predominance of pharmacological treatment documentation reflects the continued biomedical orientation of pain data systems, enabling evaluation of prescribing trends but offering limited insight into treatment safety, as few registries recorded adverse drug effects, an important omission given the escalating concerns about opioid safety and long-term pharmacotherapy outcomes.^19^ In contrast, the inconsistent inclusion of nonpharmacological modalities, such as physiotherapy, cognitive–behavioural therapies, and lifestyle interventions, suggests that multimodal pain management remains underrepresented, despite strong evidence supporting its efficacy.^50^ Notably, registries (such as CHOIR, PainData, QPR, etc.) demonstrate progress toward embedding biopsychosocial perspectives, yet this remains the exception rather than the norm. Surgical and multidisciplinary care data were similarly fragmented, constraining the capacity to examine real-world care pathways. To advance chronic pain research and clinical improvement, future registries must evolve beyond pharmacotherapy-focused models toward comprehensive, integrative datasets aligned with person-centred care principles.^61^

Economic evaluation emerged as the most underdeveloped domain despite the substantial societal and individual costs associated with persistent pain.^51^ Only a few registries (CHOIR, I-GRAINE, SQRP and ePPOC) incorporated economic variables beyond employment status, including income level, treatment costs, and productivity losses. These datasets enable assessment of the financial burden of chronic pain on patients, families, and health systems, an area often underrepresented in clinical research. However, the absence of standardized cost metrics and comprehensive economic evaluation components across registries restricts their utility for comparative analyses, evidence-based resource allocation, and informed health policy development. Integrating economic indicators within core datasets would strengthen the evidence base for resource allocation and value-based pain care.

This review offers one of the most comprehensive global assessments of chronic pain registries, systematically mapping 36 initiatives across diverse geographic and economic contexts. It extends beyond existing literature by combining a broad scoping approach with an analytical synthesis of registry characteristics, data domains, and methodological patterns. A key strength lies in its multisource data acquisition, drawing from peer-reviewed publications, official registry websites, annual reports, and direct correspondence with registry personnel, which substantially enhances accuracy and reduced reporting bias. Detailed datasets were obtained from 15 registries, allowing in-depth comparison of data structures, outcome measures, and governance frameworks. The structured data extraction and synthesis process enabled objective cross-registry analysis despite significant heterogeneity in scope and context. By combining descriptive mapping with critical interpretation, this review identifies key gaps and provides evidence-informed directions for harmonization, inclusivity, and future development of chronic pain registries worldwide.

### 4.2. Limitations and future directions

Despite these strengths, heterogeneity in data standards, pain classification, and follow-up protocols limits cross-registry comparability. Furthermore, cultural and linguistic differences may influence pain perception, reporting, care delivery, and health care utilization, introducing systemic variation in how pain and related outcomes are captured across settings. These factors pose additional challenges to the development and implementation of harmonized datasets and should be considered as a potential limitation when interpreting and comparing findings across diverse regional and international settings.

Predominant representation of tertiary-care populations restricts generalizability to primary or community settings, and inconsistent reporting of data completeness introduces potential bias. Geographic concentration in high-income regions further limits cultural and global applicability. To enhance registry utility, future initiatives should establish internationally harmonized minimal datasets that are explicitly adaptable to different cultural and linguistic contexts, expand inclusion to community and primary-care settings, and more consistently integrate economic and psychosocial dimensions. Strengthening data linkages with administrative sources may further improve registry relevance and comparability. Sustained investment, collaborative governance, and a commitment to standardized yet person-centered data frameworks are essential for realizing the full clinical and policy potential of chronic pain registries.

## 5. Conclusion

This scoping review provides a comprehensive global overview of existing chronic pain registries, revealing substantial heterogeneity in their geographic distribution, design, data domains, and methodological rigor. Although the growing number of registries, particularly since 2010, reflects increasing recognition of the value of real-world pain data, the current landscape remains fragmented, disproportionately concentrated in high-income countries, and heavily oriented toward tertiary care settings. Inconsistent adoption of standardized core datasets, limited incorporation of mechanism-based pain classification, socioeconomic indicators, and economic evaluations, and variable implementation of longitudinal follow-up constrain cross-registry comparability and translational potential. To realize the full clinical, research, and policy value of chronic pain registries, coordinated international efforts are required to develop harmonized, interoperable, and person-centred registry frameworks that extend across care settings and geographic regions. Strengthening registry infrastructure, particularly in underrepresented low- and middle-income contexts, will be critical for advancing equitable pain surveillance, informing health system planning, and improving outcomes for individuals living with chronic pain worldwide.

## Disclosures

The authors have no conflict of interest to declare.

## Supplemental digital content

Supplemental digital content associated with this article can be found online at http://links.lww.com/PR9/A433.
