# Supplementary material for: A scoping review on the characteristics of chronic pain registries
Source: Pain Rep. 2026 Aug 14;11(5):e1482. doi: 10.1097/PR9.0000000000001482 (PMC13480801; doi:10.1097/PR9.0000000000001482)
Supplement: Supplementary file 1 [file painreports-11-e1482-s001.pdf]

SUPPLEMENTARY TABLE 1: CHRONIC PAIN REGISTRY DATASET QUESTIONNAIRE

SECTION	DOMAIN	ITEMS COLLECTED
**Section 1**	**General Registry Information**	Registry name; year of establishment; geographical location; registry setting; chronic pain conditions covered; number of enrolled patients; data collection modality; population covered
**Section 2**	**Demographic Data**	Gender; age; marital status; ethnicity; education level; employment status; height/weight/BMI; geographical location; comorbidities; social benefits; disability pension; litigation
**Section 3**	**Pain Examination Details**	Pain location; pain quality; pain mechanism; diagnosis; onset/precipitating event; diurnal variation; pain duration; pain frequency; pain intensity; aggravating and relieving factors; pain interference
**Section 4**	**Patient-Reported Outcome Measures (PROMs)**	Pain intensity; pain-related disability; health-related quality of life; psychological distress; perceived self-efficacy; fatigue; sleep impact; pain catastrophizing; general health and well-being; perceived injustice; other PROMs (if applicable)
**Section 5**	**Pain Treatment Records**	Current and past pharmacological treatments; medication side effects; non-pharmacological treatments; type of healthcare professionals consulted; surgical interventions; other treatment-related data
**Section 6**	**Follow-up Assessments**	Presence of follow-up assessments (yes/no); follow-up intervals; assessments conducted at follow-up
**Section 7**	**Economic Evaluation**	Cost of treatments; cost of investigations; insurance/reimbursement impact; indirect costs; income-related data; unpaid leave
**Section 8**	**Registry Operations**	Mode of outcome data collection; specialization of registry personnel; public registry website availability; funding sources
**Section 9**	**Additional Information**	Free-text comments or additional details provided by registry representatives
